# Levels of depression, anxiety and subjective happiness among health sciences students in Croatia: a multi-centric cross-sectional study

**DOI:** 10.1186/s12888-024-05498-5

**Published:** 2024-01-13

**Authors:** Jakov Milić, Nataša Skitarelić, Dijana Majstorović, Sanja Zoranić, Marta Čivljak, Kata Ivanišević, Mario Marendić, Jasna Mesarić, Zrinka Puharić, Marijana Neuberg, Snježana Čukljek, Aleksandar Racz, Livia Puljak

**Affiliations:** 1 Croatian Carmelite Province of St. Joseph, Zagreb, Croatia; 2 Catholic Faculty of Theology, University of Zagreb, Zagreb, Croatia; 3 Department of Health Studies, University of Zadar, Zadar, Croatia; 4 Medical School, Juraj Dobrila University of Pula, Pula, Croatia; 5 Department of Nursing, University of Dubrovnik, Dubrovnik, Croatia; 6 Centre for Evidence-Based Medicine and Health Care, Catholic University of Croatia, Ilica 242, 10000 Zagreb, Croatia; 7 Faculty of Health Studies, University of Rijeka, Rijeka, Croatia; 8 University Department of Health Studies, University of Split, Split, Croatia; 9 Libertas International University, Zagreb, Croatia; 10 Department of Nursing, Bjelovar University of Applied Sciences, Bjelovar, Croatia; 11 Faculty of Dental Medicine and Health, Osijek, Croatia; 12 Department of Nursing, University North, Varaždin, Croatia; 13 University of Applied Health Sciences, Zagreb, Croatia

**Keywords:** Mental health, University students, Depression, Anxiety, Nursing, Healthcare

## Abstract

**Background:**

Previous studies have shown that symptoms of depression and anxiety were highly prevalent among health sciences students. This may lead to other professional and personal difficulties and a decrease in individuals’ well-being. This study aimed to analyze levels of depression, anxiety and subjective happiness among health sciences students in Croatia.

**Methods:**

We conducted a cross-sectional study in 10 higher education institutions in Croatia during March 2023. Eligible participants were health sciences students. Participants filled out an online survey consisting of sociodemographic questions and validated scales for determining the levels of depression (9-question Patient Health Questionnaire, PHQ-9), anxiety (General Anxiety Disorder 7-item scale, GAD-7), and happiness (Subjective Happiness Scale, SHS).

**Results:**

Of 7460 invited students, 2137 students participated in the study (29% response rate). There were 41.4% of students that exhibited at least mild depressive symptoms, with 8% of students exhibiting moderately severe symptoms and 1.8% severe depressive symptoms. Mild anxiety was found in 36.8%, moderate anxiety in 23.9% and severe anxiety in 15.8% of students. The median SHS score was 19 (15.25–22).

Women students had significantly higher levels of depression (*p* < 0.001) and anxiety (*p* < 0.001) than their men peers. Students in earlier study years showed higher levels of depression, anxiety and lower levels of subjective happiness compared to those in later study years. Students with lower self-assessed financial status had higher levels of depression (*p* < 0.001) and anxiety (*p* < 0.001). Students that failed an academic year had higher levels of depression (*p* < 0.001), but lower levels of anxiety (*p* = 0.005).

**Conclusion:**

In this study, we have shown that health sciences students exhibit high levels of depression and anxiety, at rates exceeding those in the general population reported in other studies. Our results may help educational institutions to put greater effort into the battle against mental health stigma, foster acceptance of mental health issues and encourage students to seek help when needed. Adequate mental health services are needed at universities to promote timely diagnosis and treatment of mental health problems.

**Supplementary Information:**

The online version contains supplementary material available at 10.1186/s12888-024-05498-5.

## Background

It is well documented that university students show high levels of mental distress, which is even more pronounced among health sciences students. Health sciences are often considered to be academically, psychologically, and emotionally challenging [1–4]. In our previous study conducted in 2016, we found that 60.2% of medical and health sciences students showed increased levels of depression [5]. Most research on the topic of student mental health was done on medical students. It is considered that the frequencies of mental health issues in other health sciences students are comparable to that of medical students. We have also previously shown that the levels of depressive symptoms among nursing students were comparable to those of medical students, albeit the difference was statistically significant (64.8% vs 57.3%) [5]. In the literature, the prevalence of depressive symptoms in university students varies between 8.6% and 71% [1, 3, 6–8].

Anxiety disorders are the most common psychiatric conditions in university students [9]. Medical students report symptoms of anxiety with a global prevalence rate of 33.8%, ranging between 7.7% and 65.5%, showing that a third of medical students exhibit problems with anxiety, which is much more prevalent than in the general population where it is estimated at 3–25%, depending on the instrument and study protocol [10, 11]. The prevalence of anxiety in medical students is not significantly different from that of other university students [10]. Our previous study among medical and health sciences students found high anxiety levels in 54.5% of the students, with no significant differences between medical and nursing students [5].

The prevalence of mental health issues in the population of health sciences students is alarming. These issues may lead to more severe psychiatric conditions, poor academic performance, use of harmful substances, stress-related academic dishonesty, and reduced empathy which is vital for healthcare workers [8]. Symptoms of anxiety and depression are known detrimental factors of well-being. 

Subjective happiness is one of the measures of a person’s well-being [12]. It can serve as an indicator of a person’s ability to cope with difficulties one can face. A positive outlook on life can help healthcare workers in their relationships with patients. Individuals with higher levels of happiness are shown to live longer; exhibiting happy feelings while at the workplace can make employees more productive, which is particularly important for persons working in the medical field [13].

Since our study from 2016, several changes occurred that might have led to additional increases in the levels of mental distress. Most significantly, the COVID-19 pandemic was shown to be a significant factor leading to mental health issues in the student population. Studies from 2020 have shown a higher prevalence of moderate and severe self-reported depressive and anxious symptoms in the general public and in the student populations caused by the COVID-19 pandemic [14]. A meta-analysis of the prevalence of mental health problems in the population of nursing students during the COVID-19 pandemic showed a prevalence of depression at 52%, fear at 41%, anxiety at 32%, and stress at 30% [15].

The aim of this multi-centric study was to assess the levels of depression, anxiety and subjective happiness among health sciences students in Croatia.

## Methods

### Study design

This was a multi-centric cross-sectional study.

### Reporting

The study is reported in line with the Checklist for Reporting Results of Internet E-Surveys (CHERRIES) [16].

### Participants

The study included health sciences students from 10 higher education institutions in Croatia providing health sciences studies. The following institutions participated in the study: Catholic University of Croatia, Juraj Dobrila University of Pula, Libertas International University, University of Applied Health Sciences, Polytechnic of Bjelovar, University North, University of Dubrovnik, University of Rijeka, University of Split, and University of Zadar. All health sciences students were eligible, including nursing, dental hygiene, physiotherapy, medical laboratory diagnostics, midwifery, radiological technology, occupational therapy, and sanitary engineering.

Students were asked via their official school email addresses to complete the survey hosted on SurveyMonkey. Participation was voluntary, and no incentives were offered to the students. First, information about the study was sent in a separate document with a link to the survey. The survey invitation with information about the study can be found in Supplementary file 1. Before starting the survey, participants were asked to confirm that they were giving their consent to participate in the study. Two reminders were sent, spaced one week apart, after the first email invitation. The first invitation was sent on March 6, 2023, followed by the first reminder on March 18, 2023, and the second reminder on March 20, 2023. The survey was closed on April 23, 2023. Direct identifiers of the participants were not collected.

### Ethics

The study protocol was approved by the Ethics Committees of all participating institutions. All participants gave their written informed consent in the online interface before starting the online survey. All methods were carried out in accordance with relevant guidelines and regulations.

### Survey

The students were given an online self-administered anonymous survey in Croatian language. The full text of the survey in the English language can be found in Supplementary file 2. The survey consisted of two parts. The first part included 14 questions about the participants’ sociodemographic characteristics: age, sex, university, type of study, whether they are studying full-time or part-time, school year, whether they are employed, whether they live in a rural or urban setting, the number of habitants in their place of residence, the financial status of their family and the monthly income of their family, their self-reported grade point average (GPA), whether they participated in a scientific project, and whether they failed a year in university. The second part of the survey included validated scales for determining the levels of depression, anxiety, and subjective happiness.

The Patient Health Questionnaire (PHQ-9) is a validated psychological instrument that has shown good specificity and sensitivity for detecting depressive disorders [17]. It consists of nine items that are answered on a Likert scale and correspond to one of the DSM-IV Diagnostic Criterion for symptoms of major depressive disorder [18–21]. The total score ranges from 0 to 27. Cut-off points were suggested at 5, 10, 15 and 20, corresponding to mild, moderate, moderately severe and severe levels of depressive symptoms [17]. When determining the prevalence of depressive symptoms, we used the cut-off point of 5, thus encompassing all participants with at least mild depression.

The Generalized Anxiety Disorder – 7 (GAD-7) is a validated 7-item self-report psychological instrument developed to diagnose generalized anxiety disorder. It has shown good sensitivity and specificity as a screening tool for panic disorder, social anxiety and PTSD [17]. Its seven items are answered on a Likert scale [22, 23]. The total score ranges from 0 to 21, with proposed cut-off points set at ≥ 5, ≥ 10, and ≥ 15 representing mild, moderate, and severe anxiety symptom levels, respectively [24]. When determining the prevalence of anxiety symptoms, we used the cut-off point of 5, thus encompassing all participants with at least mild anxiety.

The Subjective Happiness Scale (SHS) is a survey consisting of 4 items, and it aims to assess the respondents’ subjective happiness. Respondents are asked to characterize themselves using total ratings and ratings relative to others and to assess to what extent the characterizations of happy and unhappy individuals describe them personally. Each item is scored on a 7-point Likert scale. The possible score range is 4 to 28, with higher scores indicating a higher level of subjective happiness [25].

We used official Croatian versions of the PHQ-9 and GAD-7 since all the participants spoke Croatian. We used the Croatian translation of the Subjective Happiness Scale that our team prepared previously and used in a similar study [5]. Items were not randomized or alternated, nor was adaptive questioning applied. All the items were shown on a single page. The participants could correct their answers before sending the completed survey. Unique site visitors were not counted. The authors tested the online survey on desktop and mobile phones to ensure technical functionality before the data collection. We did not use any methods to prevent duplicate entries potentially. No surveys were submitted with an atypical timestamp.

### Statistical analysis

All surveys were included in the study, regardless of their completeness. We reported the completion rate as the number of surveys filled out and submitted divided by the number of surveys started by respondents. The distribution normality of scalar variables was tested by Kolmogorov–Smirnov and Shapiro-Wilks tests. Non-parametrical tests were used due to the non-normal distribution of all scalar variables. Numerical data were presented as medians and interquartile ranges (IQR), or as means and standard deviations. Categorical variables were presented by relative and absolute frequencies. Mann–Whitney U-test was used to assess the differences between the two groups. Cronbach’s Alpha was used to assess the internal consistency of the psychological instruments. The chi-square test was used to assess the differences in ratios between independent samples, and Spearman’s ρ was used to assess correlations among variables. The effect of multiple variables was assessed by a Stepwise Multiple Linear Regression Analysis. Data analysis was performed using an IBM SPSS Statistics version 16.0 for Windows. *P*-values less than 0.05 were considered statistically significant.

### Raw data

Raw data collected within the study, without indirect identifiers of the participants, are published on Open Science Framework (link: https://osf.io/ms2u4/).

## Results

### Participants’ characteristics

Of the 7460 invited students, 2137 students participated in the study (29% response rate). The completion rate of the survey was 97%. Response rates across institutions ranged from 16 to 65%. Response rates per institutions and number of students in different study courses accross specialty per institution are available in Supplementary file 3. One week after the first invitation, the response rate was 14% (*N* = 1022); one week after the first reminder, the response rate was 23% (*N* = 1679), finally reaching 29% after the second reminder by the time the survey was closed for further responses.

The sociodemographic characteristics of the participants are presented in Table 1. The majority of participants (86.3%) were women. The participants’ median age was 22 years (IQR 20–26). Most participants studied nursing. More than half were employed full-time. Most were employed in healthcare, lived in an urban area and had a monthly income of 1001–2000 €. Few students had ever failed a study year. Less than 20% participated in a scientific project (Table 1).

**Table 1:** Sociodemographic characteristics of the tested population (*N* = 2137)

		*n* ( %)
Sex	Men	237 (12.8)
	Women	1845 (86.3)
	Decline to answer	19 (0.9)
Institution of study	Catholic University of Croatia	187 (8.7)
	Libertas International University	130 (6.1)
	Juraj Dobrila University of Pula	193 (9.0)
	University North	194 (9.1)
	University of Applied Health Sciences	542 (25.4)
	University of Dubrovnik	47 (2.2)
	University of Rijeka	291 (13.6)
	University of Split	260 (12.2)
	University of Zadar	153 (7.2)
	Polytechnic of Bjelovar	140 (6.5)
Type of study	Nursing	1479 (69.2)
	Clinical nutritionism	3 (0.1)
	Dental hygiene	1 (0.0)
	Physiotherapy	332 (15.5)
	Medical laboratory diagnostics	74 (3.5)
	Midwifery	44 (2.1)
	Radiological technology	101 (4.7)
	Occupational therapy	33 (1.5)
	Sanitary engineering	68 (3.2)
	Did not specify	3 (0.1)
Full-time or part-time student	Full-time	1123 (52.5)
	Part-time	1014 (47.4)
Year of study	1	198 (9.3)
	2	527 (24.6)
	3	203 (9.5)
	4	594 (27.8)
	5	615 (28.8)
Employment	Employed in the medical field	821 (38.4)
	Employed outside the medical field	424 (19.8)
	Unemployed	892 (41.7)
Residence	Urban	1451 (67.9)
	Rural	686 (32.1)
Size of residence	< 10000	879 (41.1)
	10001–50000	545 (25.5)
	50001–100000	226 (10.6)
	100001–200000	136 (6.4)
	> 200000	351 (16.4)
Self-assessed financial status	1 – very low	16 (0.7)
	2	105 (4.9)
	3	1007 (47.1)
	4	826 (38.7)
	5—excellent	183 (8.6)
Average monthly income	< 1000 €	193 (9.0)
	1001–2000 €	905 (42.3)
	2001–3000 €	636 (29.7)
	> 3000 €	403 (18.8)
Failing a year in college	Yes	156 (7.3)
	No	1981 (92.7)
Doing a scientific project	Yes	404 (18.9)
	No	1733 (81.1)

### Levels of depression, anxiety and subjective happiness among health sciences students

All scalar variables showed a deviation from normal distribution as tested by the Kolmogorov–Smirnov test. Cronbach’s Alpha test showed satisfactory internal consistency of the instruments used in the study, with PHQ-9 having an alpha of 0.905, GAD-7 of 0.905 and SHS of 0.807. Suicidal or auto-destructive ideations were present in 414 (19.4%) of students.

The median scores were as follows: PHQ-9 8 (4–13), GAD-7 7 (4–12), SHS 19 (15.25–22), and GPA 4 (4–4). There were no significant differences in GPA between the sexes (*p* = 0.064).

Differences in PHQ-9, GAD-7 and SHS scores are presented in Table 2. Significant differences in the levels of depression were found based on sex (*p* < 0.001), whether the students worked part-time or full-time (*p* = 0.007), year of study (*p* < 0.001), employment (*p* < 0.001), self-assessed financial status (*p* < 0.001), average monthly income (*p* < 0.001), failing a year in college (*p* < 0.001). Significant differences in the anxiety levels were found based on sex (*p* < 0.001), year of study (*p* < 0.001), employment (*p* < 0.001), self-assessed financial status (*p* < 0.001), average monthly income (*p* = 0.010), failing a year in college (*p* = 0.005). Significant differences in the subjective happiness scores were found based on whether the students worked part-time or full-time (*p* = 0.019), year of study (*p* < 0.001), employment (*p* < 0.001), self-assessed financial status (*p* < 0.001), average monthly income (*p* < 0.001), failing a year in college (*p* < 0.001).

**Table 2:** Medians and interquartile ranges (IQR) of the scores of the PHQ-9 (The patient health questionnaire-9), GAD-7 (Generalized Anxiety Disorder-7), and Subjective Happiness Scale (SHS) scales depending on the different variables and the difference between those groups. For small groups, the 75th percentile, or both the 25th and the 75th percentile could not be calculated (*N* = 2138 participants)

		PHQ-9		GAD-7		SSH	
		median (IQR)	*p*	median (IQR)	*p*	median (IQR)	*p*
Sex	Men	3 (7–10.5)	< 0.001	3 (6–9)	< 0.001	20 (16–23)	0.069
	Women	8 (4–14)		8 (4–12)		19 (15–22)	
Type of study	Nursing	8 (4–13)	0.222	7 (4–12)	0.072	19 (16–22)	0.156
	Clinical nutritionism	12 (1)		8 (5)		14 (13)	
	Dental hygiene	18		14		25	
	Physiotherapy	7.5 (3.25–13)		7 (3–11)		19 (15–23)	
	Medical laboratory diagnostics	9 (5–15.25)		10 (4.75–14.25)		17 (15–21)	
	Midwifery	9.5 (4.25–12.75)		10 (5–14.75)		19 (16.75–24)	
	Radiological technology	8 (3.5–14)		7 (4–12)		18 (14–22)	
	Occupational therapy	8 (6–13)		8 (3.5–12.5)		20 (15.25–24)	
	Sanitary engineering	9 (6–15)		8.5 (5–12)		17 (14–21)	
Full-time or part-time student	Full-time	8 (4–14)	0.007	8 (4–13)	0.061	19 (15–22)	0.019
	Part-time	8 (4–13)		7 (4–12)		19 (16–22)	
Year of study	1	9 (5–14)	< 0.001	8 (4–12)	< 0.001	14 (12–23)	< 0.001
	2	9 (4–14)		8 (4–13)		19 (15–22)	
	3	8 (4–14)		9 (5–13)		18 (15–22)	
	4	6 (3–11)		6 (3–10)		20 (17–24)	
	5	6 (3–11)		6 (4–9)		20 (17–23)	
Employment	In the medical field	7 (4–12)	< 0.001	7 (4–11)	< 0.001	20 (17–22)	< 0.001
	Outside the medical field	9 (5–16)		8.5 (5–13)		18 (14–22)	
	Unemployed	8 (4–14)		8 (4–12)		19 (15–22)	
Residence	Urban	8 (4–13)	0.888	7 (4–12)	0.793	19 (16–22)	0.083
	Rural	8 (4–14)		7 (4–12)		19 (15–22)	
Size of residence	< 10000	8 (4–14)		7 (4–12)		19 (15–22)	
	10001–50000	9 (4–13)	0.650	8 (4–12)	0.628	19 (16–22)	0.178
	50001–100000	8 (4–13)		7 (4–11.25)		19 (16–23)	
	100001–200000	7 (3–13.75)		7 (3–12)		19 (15–22.25)	
	> 200000	8 (4–13)		8 (4–13)		20 (15–22)	
Self-assessed financial status	1	15 (4.25–23.75)	< 0.001	8.5 (3–14.75)	< 0.001	15.5 (13.75–22.25)	< 0.001
	2	12 (6–18.75)		11 (6–15)		16.5 (13–20)	
	3	9 (5–14)		8 (5–12)		18 (15–22)	
	4	7 (3–13)		7 (4–11.25)		20 (16–23)	
	5	6 (3–11)		6 (3–12)		21 (17–24)	
Average monthly income	< 1000€	9 (6–16)	< 0.001	9 (5–14)	0.010	18 (15–22)	< 0.001
	1001–2000€	9 (4–14)		8 (5–12)		18 (15–22)	
	2001–3000€	7 (4–13)		7 (4–12)		19 (16–23)	
	> 3000€	7 (3–13)		7 (4–12)		20 (17–23)	
Failing a year in college	Yes	11 (5–16)		7 (4–12)		17 (14–21)	
	No	8 (4–13)	< 0.001	9.5 (5–14)	0.005	19 (16–22)	< 0.001
Doing a scientific project	Yes	8 (4–14)		8 (5–13)	0.105	19 (15–22)	
	No	8 (4–13)	0.184	7 (4–12)		19 (16–22)	0.783

There was a strong positive correlation between depression and anxiety (rho = 0.826, *p* < 0.001), and those variables had a strong negative correlation with subjective happiness (depression: rho = -0.600, *p* < 0.001; anxiety, rho = -0.556, *p* < 0.001). Age negatively correlated with depression (-0.145, *p* < 0.001) and anxiety (rho = -0.091, *p* < 0.001) and positively with subjective happiness (rho = 0.083, *p* < 0.001).

Since the scores of the PHQ-9 and GAD-7 surveys can be divided into categories based on predefined cut points, the frequencies of those categories are presented in Table 3. Men had higher frequencies of low and mild anxiety, while women had higher frequencies of moderate and severe anxiety (*p* < 0.001). Men had higher frequencies of low depressive symptoms, while women had higher frequencies of mild, moderate, moderately severe and severe depressive symptoms (*p* = 0.003).

**Table 3:** The frequencies and percentages of the categories of depressive and anxiety symptoms according to the GAD-7 (Generalized Anxiety Disorder-7) and PHQ-9 (The patient health questionnaire-9) scales and the sex-related differences (*N* = 2138)

		n (%)			*P* (Chi square)
		**Men**	**Women**	**Total**	< 0.001
GAD-7	Low anxiety	81 (32.5)	398 (22.4)	484 (23.6)	
	Mild anxiety	103 (41.4)	646 (36.3)	752 (36.8)	
	Moderate anxiety	41 (16.5)	445 (25.0)	488 (23.9)	
	Severe anxiety	24 (9.6)	291 (16.3)	324 (15.8)	
PHQ-9	Low levels of depressive symptoms	189 (69.2)	1056 (57.2)	1253 (58.6)	0.003
	Mild depressive symptoms	42 (15.4)	383 (20.8)	427 (20.0)	
	Moderate depressive symptoms	26 (9.5)	216 (11.7)	246 (11.5)	
	Moderately severe depressive symptoms	15 (5.5)	152 (8.2)	172 (8.0)	
	Severe depressive symptoms	1 (0.4)	38 (2.1)	39 (1.8)	

Note: 19 participants declined to specify their gender and were excluded from the analysis. Due to missing data in certain items of the GAD-7, the scores could not be calculated for 90 participants

Stepwise Multiple Linear Regression Analysis determined how different variables affected the PHQ-9, GAD-7 and SHS scores. Variables that were offered to the models were as follows: gender, age, type of study, GPA, year of study, full-time or part-time student, employees yes/no, residence, self-assessed financial status, average monthly income, failing a year in college yes/no, previous research project yes/no.

All variables offered to the models for PHQ-9, GAD-7 and SHS were included in the final models. Gender, monthly income and failing a year were the most significant predictors for all the tested variables.

The model for PHQ-9 explained 8.4% of the variance, determined as an adjusted R squared (R^2^) with a standard error of 6.28. The ANOVA test results suggest satisfactory explanatory power, F = 16.17, df = 12, *p* < 0.001 (Table 4).

**Table 4:** The unstandardized coefficient betas of the variables included in the stepwise multiple linear regression analysis

	**PHQ-9**		**GAD-7**		**SHS**	
	Beta	Standard Error	Beta	Standard Error	Beta	Standard Error
Gender	2.042^***^	0.411	2.065^***^	0.343	-0.613^*^	0.310
Age	-0.138^***^	0.025	-0.092^***^	0.021	0.067^***^	0.019
Type of study	0.051	0.065	-,023	0.055	-0.069	0.049
GPA	-0.889^**^	0.286	-0.491^*^	0.239	0.556^*^	0.217
Year of study	-0.347^**^	0.130	-0.246^*^	0.108	-0.113	0.099
Full-time or part-time student	-0.288	0.379	-0.277	0.316	0.248	0.286
Employed	-0.176	0.224	-0.072	0.187	-0.288	0.170
Residence	-0.332	0.296	-0.149	0.248	-0.178	0.224
Financial status	-0.079	0.174	-0.027	0.145	0.214	0.131
Monthly income	-1.452^***^	0.207	-0.887^***^	0.173	1.177^***^	0.157
Failing a year in college	2.041^***^	0.536	1.189^**^	0.448	-1.200^**^	0.405
Previous research project	0.834^*^	0.356	0.776^**^	0.297	-0.040	0.270

^*^p < 0.05

^**^p < 0.01

^***^p < 0.001

The model for GAD-7 explained 5.8% of the variance, determined as an adjusted R squared (R^2^) with a standard error of 5.24. The ANOVA test results suggest satisfactory explanatory power, F = 10.82, df = 12, *p* < 0.001 (Table 4).

The model for SHS explained 6.8% of the variance, determined as an adjusted R squared (R^2^) with a standard error of 4.7. The ANOVA test results suggest satisfactory explanatory power, F = 12.46, df = 12, *p* < 0.001 (Table 4).

## Discussion

Our analysis of the levels of depression, anxiety and subjective happiness in students of health sciences students in Croatia showed concerning results. There were 41.4% of students that exhibited at least mild depressive symptoms, with 8% of students exhibiting moderately severe symptoms, and 1.8% severe depressive symptoms. Mild anxiety was found in 36.8%, moderate anxiety in 23.9% and severe anxiety in 15.8% of students.

Women students had significantly higher levels of depression and anxiety than their men peers. Suicidal or auto-destructive ideations were present in 19.4% of students. Students in earlier years of the study showed higher levels of anxiety, depression and lower levels of subjective happiness. Students with lower self-assessed financial status had higher levels of anxiety and depression. Students that failed an academic year had higher levels of depression but lower levels of anxiety.

In our previous study conducted in 2016, we assessed the same variables in medical and nursing students [5]. We found that the anxiety levels in the current study were markedly higher than in our previous study. Among nursing students, we previously found that 55.7% exhibited at least mild anxiety levels, compared to 76.4% in this study. On the contrary, the levels of depression were lower in the current research; 57.3% of students exhibited at least mild depressive symptoms in our previous research, compared to the current 41.4% [5]. However, the results are not completely comparable since, in the current study, we included other health science university studies and multiple institutions from different parts of Croatia. Thus, the sample was more diverse in the current study.

Pursuing higher education can feel overwhelming for many students, marking a period of first independence from their parents, often accompanied by financial burdens, dealing with long study hours and pressure from family members [26]. Indeed, we found that low self-assessed financial status was associated with higher levels of depression and anxiety in our study. Kumar et al. found that higher financial status was associated with higher levels of happiness in nursing students [27].

Many students have to work part-time or full-time to ease the financial burden of university life, with the share of students working reaching up to 85% in certain countries [28, 29]. It has been shown that working students have more prevalent physical and mental problems due to the increased workload, sleep deprivation and lack of social contact with loved ones [29]. In our study, students that worked showed higher levels of depression and anxiety and lower levels of subjective happiness compared to students that did not work. When comparing these variables based on the type of employment, it can be seen that between the groups of students working in the medical field, outside the medical field, and those who are unemployed, students working in the medical field showed the lowest levels of depression and anxiety, and those who were employed outside the medical field, the highest. These results may imply that having a job in the medical field may give the students a sense of stability and competence, which may lead to better mental health.

Higher levels of depression and anxiety in students of lower years of study might be associated with balancing new duties, but the curriculum obligations must also be taken into account in future research. Bachelor studies are often considered to be more difficult than Master’s studies in Croatia, and the analysis of whether this is a factor in students’ levels of depression and anxiety should be expanded upon in the future since several studies found that school was the main stressor for students [8, 30]. Medical and health sciences are often considered more demanding than other types of studies due to difficult courses but also because of the clinical work, which is often emotionally and physically demanding. Medical students have shown higher levels of depression and burnout than other university types of studies, and most research on the topic of mental health of university students was done on this population. Studies have shown that medical students often exhibit symptoms of burnout, with prevalence as high as 70—95% [8, 31–33]. The prevalence of depression in this population is 10–15% higher than in the general population [30].

In our study, we found that students that failed a year in college had higher levels of depression and anxiety. However, it is difficult to suggest, based on our data, the direction in which these variables are associated, i.e., whether the initially decreased mental health leads to problems with studying or whether the stress related to school responsibilities leads to decreased mental health. The most plausible answer is that there is an interplay between the variables that eventually form a vicious circle.

Even though the levels of depression are higher in medical students than in other health sciences students, the results are comparable, and there are no significant differences in anxiety levels between medical students and other health science students [5, 10]. A study in Turkey found that nursing students had a borderline to high prevalence of mental health problems compared to other university types of studies and the general population [34]. Other studies found that nursing and public health students were less likely to have mental health problems than other university majors [35]. In the current study, we found a prevalence of at least mild depressive symptoms of 41.4%, which is similar to previous studies [1, 3, 5–8, 36].

Research has shown that 6–26% of students are diagnosed with a mental health issue [1, 7, 8, 30]. Studies have shown that anxiety is more prevalent than depression, which is in line with our results [30]. Women students are usually more affected [37–40], even though a recent meta-analysis found no gender-related differences in anxiety in the medical student population [10]. In our research, we also found significantly higher levels of anxiety in women students. It is important to note that most mental health problems occur in early adulthood, with the onset of 75% of mental health problems occurring by the age of 25, which coincides with the age of pursuing higher education [2]. Still, only a third of them seek treatment [8, 41]. This shows that the stigma of mental health issues still exists, even among health sciences students [42]. Stigma is considered the most important obstacle to seeking professional help [2].

It has been shown that untreated mental health issues may progress into more complex psychiatric disorders, school dropout, addiction, and other auto-destructive behaviors [43]. However, things might be changing as more students seek help at a rate exceeding enrolment increases [43]. Not requesting help may lead to other negative outcomes, such as school dropout, suicidal ideation and burnout [41]. It is known that medical professionals have high rates of suicide [26, 32]. Medical students show rates of suicidal ideations of 7.4 -24.2%, which are higher than those in the general university student population which are at a rate of 6.7% [3, 9, 41, 43]. Even though we did not ask our participants about their suicidal ideations, we asked them about their self-destructive and suicidal ideations (Item 9 from the PHQ-9 scale: “*Thoughts that you would be better off dead, or of hurting yourself*”), and we found a rate of 19.4% which is comparable to prior results about suicidal ideations.

Studies on nursing students showed that during the COVID-19 pandemic, they showed higher levels of anxiety or depression [15, 36, 38, 44–47]. Previous research has also shown that in pandemic outbreaks, nurses are more likely to experience worse mental health than doctors, which might be related to the increased time nurses spend in contact with patients when compared to physicians [44]. The COVID-19 pandemic has put a unique strain on students. In Croatia, following the onset of the COVID-19 pandemic, students were abruptly switched to online-only education, and not all students had a positive attitude towards that switch [48].

Meda et al. conducted a study on Italian students and reported higher levels of depression during the COVID-19 pandemic, with those students who previously showed no problems with mental health having a more pronounced increase in their levels of depression [49]. However, the same research has shown that after the lockdown, the prevalence of psychiatric issues returned to levels before the lockdown [49].

Our study was conducted during March–April 2023 when most measures against COVID-19 were abolished, except in the clinical setting. Our results are in line with those of Meda et al., as the levels of depression and anxiety are comparable to those of similar research done before the COVID-19 pandemic [5, 49].

Studies have shown that higher mental well-being is positively associated with empathy [40]. Empathy is one of the most important traits for workers in healthcare. In this study, we assessed subjective happiness as a measure of well-being [12]. Previous studies have shown that nursing students show higher levels of subjective happiness when compared to medical students [5]. It is known that happier individuals tend to live longer, are more productive at the workforce and contribute to making society a better place through socially cooperative roles such as voluntary work [13]. Previous, albeit limited, research has shown low-to-moderate happiness levels in the nursing student population [27].

High levels of mental health problems in the population of students of health sciences present a reason for concern as it may damage professional performance, decrease empathy, ethical conduct and professionalism, and it may lead to personal consequences such as substance abuse, broken relationships, and suicidal ideation [1, 50].

### Limitations of the study

Our study has several limitations. It was an online survey that guaranteed confidentiality and the students were free to decide whether they wished to participate. Due to our response rate (29%), it is possible that the students who exhibit mental problems were overrepresented and more motivated to participate in the study due to self-selection bias [51]. Since we used the cut-off points for mild anxiety and depression, the prevalence we found could represent an overestimation. Our study may provide a baseline for monitoring students’ mental health in the future. Even though we aimed to use a limited set of instruments to have a better completion rate, a broader set of instruments would give us a better picture of the possible underlying causes of the problems, and a qualitative study of these findings would be beneficial. Variables that would be useful to assess in future research are the levels of burnout, empathy, stress and personality and whether the students are diagnosed with a mental condition and are seeking professional help. Due to the nature of the study, our findings do not represent the official diagnoses of participants. Also, we would like to emphasize that even though there were more women among participants, this is representative of the demographics of health sciences students.

## Conclusion

This study shows that health science students exhibit high levels of depression and anxiety at rates exceeding those in the general population reported in other studies. Since the well-being of medical professionals is essential for their professional work, adequate care must be given to these individuals to prevent further progression of mental illness.

Our results may help educational institutions to put greater effort into the battle against mental health stigma, foster acceptance of mental health issues and encourage students to seek help when needed. Adequate mental health services are needed at universities to promote timely diagnosis and treatment of mental health problems.

### Supplementary Information

**Additional file 1: Supplementary file 1.** Invitation and information for participants.

**Additional file 2: Supplementary file 2.** Survey used in the research.

**Additional file 3: Supplementary file 3.** Response rates per institutions and number of students in different study courses accross specialty per institution.

## Data Availability

Raw data collected and analyzed within this study are published on Open Science Framework (link: https://osf.io/ms2u4/), except for the indirect identifiers of the participants.

## Abbreviations

PHQ-9: Patient Health Questionnaire 9
GAD-7: General Anxiety Disorder 7
SHS: Subjective Happiness Scale
GPA: Grade point average
IQR: Inter-quartile range
