# Supplementary material for: Levels of depression, anxiety and subjective happiness among health sciences students in Croatia: a multi-centric cross-sectional study
Source: BMC Psychiatry. 2024 Jan 13;24:50. doi: 10.1186/s12888-024-05498-5 (PMC10787412; doi:10.1186/s12888-024-05498-5)
Supplement: Supplementary file 1 — Additional file 1: Supplementary file 1. Invitation and information for participants. [file 12888_2024_5498_MOESM1_ESM.docx]

**Supplementary file 1:** **Invitation and information for participants**

**E-mail invitation to participate in the study**

**Message title:** Invitation to participate in the study on depression, anxiety and happiness of students

**Message text:**

Dear and respected colleagues,

Hello, I hope you are well and that you might be willing to participate in the study conducted throughout Croatia among health sciences students. Our study conducted in 2016, in which we examined the frequency of depressive and anxiety symptoms among medical and nursing students in Croatia, showed that as many as 55% of students had anxiety symptoms, and 60% of students had symptoms of depression. These are worrying data that prompted us to conduct such a study again, this time in the multiple institutions across Croatia.

The results of this study will better help us design procedures to support the well-being and health of our students. Therefore, your opinion and participation are very important to us.

More detailed information about the study can be found in the attachment.

The study is conducted through an anonymous online survey, and the average time to complete the questionnaire is about 5 minutes.

Link to access the survey:

*https://www.surveymonkey.com/r/8T8SNYF*

Thank you in advance for your cooperation and sincere regards,

[*first and last name of the co-author from the institution*]

**Attachment: Information about the study**

**Study title: Levels of depression, anxiety and subjective happiness among health sciences students in Croatia: a multi-centric cross-sectional study**

Dear Sir/Madam,

You are invited to participate in the study on the frequency and severity of anxiety and depressive symptoms and the happiness level among health sciences students in the Republic of Croatia. This document will provide information intended to help you decide whether you want to participate in this study. Before you decide, we want to explain why this study is being conducted and what it involves.

The aim of this study is to examine the basic characteristics of students with a survey and to examine the level of anxiety, depression and happiness among students with standard validated questionnaires. The examination will be conducted at several universities and polytechnic institutions in Croatia, where health workers of various profiles are trained. The data we get through this study can help us design new forms of support for students if needed.

It is up to you to decide whether you want to participate or not. The study is conducted through an online survey lasting about 5 minutes. If you decide to participate, on the first page of the survey, you can express your consent to participate. Your decision to participate in this study is voluntary, and you can withdraw freely and without any consequences at any time without giving a reason.

Under applicable law, you have the right to access your personal data, correction, deletion, limitation of processing and portability of personal data, and the right to object to processing and lodge a complaint with the Personal Data Protection Agency. We can delete your data from the research database if you decide to do so before the publication of the results.

Researchers will follow the procedure for protecting personal data in medical research. All questionnaires will be categorized exclusively under a numerical code, and nowhere on the questionnaire will you be asked to provide your first and last name or e-mail address. You will be invited to participate in the study by a teacher at your institution who will not share your name and/or contact with other research team members.

The collected data will be used only for the study in question and will be stored in digital form on the computer of the principal investigator (Prof. Livia Puljak ), protected by a password. Data will be stored for at least five years after the end of data collection. The researchers participating in the study will have access to the data during the study. The results will be used to improve the quality of teaching, writing scientific articles and lectures.

There are no risks associated with this study; that is, the level of discomfort in this study is no greater than what you experience in everyday life situations. A potential benefit for students from participating in this study is that, based on the study results, the need to introduce additional support for students will be considered.

The study was reviewed by the Ethics Committee of the Croatian Catholic University, which approved the study protocol after reviewing the complete documentation. The study is conducted following the guidelines for the safety of persons participating in such study, including the *Declaration of Helsinki*.

If you would like to be informed about the results after the study has been completed, or if you have any questions regarding the study, please contact the principal investigator: prof. Livia Puljak MD, PhD; e-mail: livia.puljak@unicath.hr; phone: + 385 (0) 1 370 66 33.

If you have any complaints about the study or how the data were collected, please contact the principal investigator: prof. Livia Puljak MD, PhD; e-mail: livia.puljak@unicath.hr; phone: + 385 (0) 1 370 66 33.

Thank you for reading this document and considering participating in this study.

Prof. Livia Puljak MD, PhD

Principal investigator

e-mail: livia.puljak@unicath.hr

phone: + 385 (0) 1 370 66 33
