# Supplementary material for: Levels of depression, anxiety and subjective happiness among health sciences students in Croatia: a multi-centric cross-sectional study
Source: BMC Psychiatry. 2024 Jan 13;24:50. doi: 10.1186/s12888-024-05498-5 (PMC10787412; doi:10.1186/s12888-024-05498-5)
Supplement: Supplementary file 2 — Additional file 2: Supplementary file 2. Survey used in the research. [file 12888_2024_5498_MOESM2_ESM.docx]

**Supplementary file 2. Survey used in the research**

**1**. How old are you? _______

**2**. Sex

a. Man

b. Woman

c. I decline to answer

**3**. Type of studies:

a. Dental hygiene

b. Physiotherapy

c. Medical laboratory diagnostics

d. Midwifery

e. Radiological technology

f. Occupational therapy

g. Sanitary engineering

h. Nursing

i. Other, specify:_________

**4.** Year of study:

1st

2nd

3rd

4th (1st year of Master’s study)

5th (2nd year of Master’s study)

**5** . Place of residence:

a. City

b. Village

**6.** Rate your family's financial status from 1 to 5, where 1 means very bad and 5 means excellent:

1 2 3 4 5

**7.** Your current grade point average rounded to two decimal places: ______

**8.** Did you repeat the year at least once during your studies?

a. Yes

b. No

**9.** Did you participate in scientific research during your studies?

a. Yes

b. No

| During **the past 2 weeks,** how often did any of the following problems cause you difficulty?  Circle your answer in the table. The rating scale is as follows:  0 - Not at all 1 - Several days 2 - More than half the days 3 - Nearly every day |
| --- |

| 10. Feeling nervous, anxious or on edge | 0 | 1 | 2 | 3 |
| --- | --- | --- | --- | --- |
| 11. Not being able to stop or control worrying | 0 | 1 | 2 | 3 |
| 12. Worrying too much about different things | 0 | 1 | 2 | 3 |
| 13. Trouble relaxing | 0 | 1 | 2 | 3 |
| 14. Being so restless that it's hard to sit still | 0 | 1 | 2 | 3 |
| 15. Becoming easily annoyed or irritable | 0 | 1 | 2 | 3 |
| 16. Feeling afraid as if something awful might happen | 0 | 1 | 2 | 3 |
| 17. Little interest or pleasure in doing things? | 0 | 1 | 2 | 3 |
| 18. Feeling down, depressed, or hopeless? | 0 | 1 | 2 | 3 |
| 19. Trouble falling or staying asleep, or sleeping too much? | 0 | 1 | 2 | 3 |
| 20. Feeling tired or having little energy? | 0 | 1 | 2 | 3 |
| 21. Poor appetite or overeating? | 0 | 1 | 2 | 3 |
| 22. Feeling bad about yourself — or that you are a failure or have let yourself or your family down? | 0 | 1 | 2 | 3 |
| 23. Trouble concentrating on things, such as reading the newspaper or watching television? | 0 | 1 | 2 | 3 |
| 24. Moving or speaking so slowly that other people could have noticed? Or so fidgety or restless that you have been moving a lot more than usual? | 0 | 1 | 2 | 3 |
| 25. Thoughts that you would be better off dead, or thoughts of hurting yourself in some way? | 0 | 1 | 2 | 3 |

| **For each of the above statements or questions, choose the number on the scale that you think best describes you.** | |
| --- | --- |
| 26 | In general, I consider myself:  not a very happy person  1 2 3 4 5 6 7 a very happy person |
| 27 | Compared with most of my peers, I consider myself:  less happy 1 2 3 4 5 6 7 more happy |
| 28 | Some people are generally very happy.  They enjoy life regardless of what is going on, getting the most out of everything.  To what extent does this characterization describe you?  not at all 1 2 3 4 5 6 7  a great deal |
| 29 | Some people are generally not very happy.  Although they are not depressed, they never seem as happy as they might be.  To what extent does this characterization describe you?  not at all 1 2 3 4 5 6 7 a great deal |
