# Supplementary material for: Levels of depression, anxiety and subjective happiness among health sciences students in Croatia: a multi-centric cross-sectional study
Source: BMC Psychiatry. 2024 Jan 13;24:50. doi: 10.1186/s12888-024-05498-5 (PMC10787412; doi:10.1186/s12888-024-05498-5)
Supplement: Supplementary file 3 — Additional file 3: Supplementary file 3. Response rates per institutions and number of students in different study courses accross specialty per institution. [file 12888_2024_5498_MOESM3_ESM.docx]

**Supplementary file 3. Response rates per institutions and** **number of students in different study courses accross specialty per institution**

**Response rates per institutions**

| **Institution name** | **Number of students** | **Number of participants** | **Response rate** |
| --- | --- | --- | --- |
| Catholic University of Croatia | 422 | 187 | 44.3 |
| Libertas International University | 391 | 130 | 33.2 |
| Juraj Dobrila University of Pula | 296 | 193 | 65.2 |
| University North | 700 | 194 | 27.7 |
| University of Applied Health Sciences | 3415 | 542 | 15.9 |
| University of Dubrovnik | 78 | 47 | 60.3 |
| University of Rijeka | 733 | 291 | 39.7 |
| University of Split | 708 | 260 | 36.7 |
| University of Zadar | 227 | 153 | 67.4 |
| Polytechnic of Bjelovar | 490 | 140 | 28.6 |
| **Total** | **7460** | **2137** | **28.6** |

**Number of students in different study courses accross specialty per institution**

| **Institution name** | **Number of students** | **Type of study course per speciality** |
| --- | --- | --- |
| Catholic University of Croatia | 422 | 422 nursing students |
| Libertas International University | 391 | 391 physiotherapy students |
| Juraj Dobrila University of Pula | 296 | 296 nursing students |
| University North | 700 | 700 nursing |
| University of Applied Health Sciences | 3415 | 1568 nursing  926 physiotherapy  257 medical laboratory diagnostics  280 sanitary engineering  239 radiological technology  145 occupational therapy |
| University of Dubrovnik | 78 | 78 nursing students |
| University of Rijeka | 733 | 295 nursing students  90 midwifery  219 physiotherapy  65 radiological technology  47 promotion of mental health  17 clinical nutritionism |
| University of Split | 708 | 480 nursing students  62 midwifery  175 physical therapy  107 radiological technology  44 medical laboratory diagnostics |
| University of Zadar | 227 | 227 nursing students |
| Polytechnic of Bjelovar | 490 | 490 nursing students |
| **Total** | **7460** |  |
